# Alexithymia may explain the genetic relationship between autism and sensory sensitivity

**DOI:** 10.1038/s41398-025-03254-1

**Published:** 2025-03-05

**Authors:** Isabel Yorke, Jennifer Murphy, Fruhling Rijsdijk, Emma Colvert, Stephanie Lietz, Francesca Happé, Geoffrey Bird

**Affiliations:** 1 Department of Child and Adolescent Psychiatry, Institute of Psychiatry, Psychology and Neuroscience, King’s College London, London, UK; 2 Department of Psychology, University of Surrey, Guildford, UK; 3 Department of Psychology, Anton de Kom University, Paramaribo, Suriname; 4 Social, Genetic and Developmental Psychiatry Centre, Institute of Psychiatry, Psychology and Neuroscience, King’s College London, London, UK; 5 Department of Experimental Psychology, University of Oxford, Oxford, UK; 6 Centre for Research in Autism and Education, Institute of Education, University College London, London, UK

**Keywords:** Autism spectrum disorders, Human behaviour, Clinical genetics

## Abstract

Sensory symptoms are highly prevalent amongst autistic individuals and are now considered in the diagnostic criteria. Whilst evidence suggests a genetic relationship between autism and sensory symptoms, sensory symptoms are neither universal within autism nor unique to autism. One explanation for the heterogeneity within autism and commonality across conditions with respect to sensory symptoms, is that it is alexithymia (a condition associated with difficulties identifying and describing one’s own emotions) that has a genetic relationship with sensory symptoms, and that alexithymia commonly co-occurs with autism and with several other conditions. Using parent-reports of symptoms in a sample of adolescent twins, we sought to examine the genetic association between autism, alexithymia and sensory symptoms. Results showed that the genetic correlation between autism and sensory symptoms was not significant after controlling for alexithymia. In contrast, after controlling for variance in alexithymia explained by autism, the genetic correlation between alexithymia and sensory symptoms was significant (and the proportion of variance explained by genetic factors remained consistent after controlling for autism). These results suggest that 1) alexithymia and sensory symptoms share aetiology that is not accounted for by their association with autism and 2) that the genetic association between sensory symptoms and autism may be, in part or wholly, a product of alexithymia. Future research should seek to examine the contribution of alexithymia to sensory symptoms across other conditions.

## Background

Sensory sensitivities, both hyper- and hypo-sensitivity, are reported by 45–95% of autistic^1^ individuals [2–6]. Reported across almost all sensory domains (e.g., taste, touch, audition, smell, vision and interoception; [3, 5, 7]), atypical sensory experience is now considered a diagnostic feature of autism [8], has been linked to physical and mental health conditions that co-occur with autism (e.g., anxiety; [9, 10]), and ranks as one of the top concerns reported by autistic individuals [11]. Given the impact of sensory sensitivity on the wellbeing of autistic individuals, understanding the aetiological factors that contribute towards atypical sensory experience is an urgent research goal.

Quantitative genetic studies of autism, exploiting the differing genetic relationships between monozygotic (MZ) and dizygotic (DZ) twins, have demonstrated up to 95% heritability for autism [12]. However, both behavioural and genetic evidence converge to suggest that the constellation of symptoms constituting autism (social and non-social) cannot be explained by a unitary cause [13–15]. In terms of sensory symptoms, evidence from twin studies suggest sensory symptoms are heritable [16–18] and that moderate-strong genetic associations are observed between autistic traits and sensory symptoms, suggesting they share a genetic, as well as phenotypic, relationship [18, 19]. Sensory symptoms are also reported at higher rates in the relatives of autistic individuals, suggesting they are part of the broader autism phenotype [20, 21].

Whilst the above evidence points to a shared genetic basis for autism and sensory symptoms, it is important to note that sensory symptoms are neither universal, nor unique, to autism [22]. There is evidence that the presence/absence and pattern of symptoms could constitute specific subtypes across autistic individuals [23], and sensory symptoms are also highly prevalent in other conditions such as Attention-Deficit Hyperactivity Disorder (ADHD; [24]) and Schizophrenia [25]. Whilst evidence points to genetic overlap between autism and sensory symptoms, the explanation for the heterogeneity within autism, and presence of sensory symptoms across conditions, remains under-examined.

One potential explanation for the heterogeneity within autism, and presence of sensory symptoms across conditions, is that it is alexithymia that has a genetic relationship with sensory symptoms, and that alexithymia is common within the autistic population and within several other conditions. Alexithymia is a condition associated with difficulties identifying and describing one’s own emotions [26] that is to some extent heritable (~30–45%; [27–29]) and is highly prevalent in autistic individuals (40–65%) compared to non-autistic individuals (5–10%; [30, 31]. Alexithymia is also highly prevalent across several other conditions including eating disorders, anxiety and depression, suggesting that alexithymic features may be considered a ‘transdiagnostic risk-factor’ [7]. Importantly, however, alexithymia is dissociable from autism and other conditions [32]; not all autistic individuals experience co-occurring alexithymia, and vice versa, meaning one is neither necessary nor sufficient for the other.

Whilst the severity of alexithymia (and not autism symptoms) has been found to explain variance in emotion recognition [33, 34], empathy [35] and eye gaze [36], suggesting that alexithymia may contribute towards the social features of autism, there is good reason to expect that alexithymia may contribute towards non-social features, particularly sensory symptoms. Whilst multiple mechanisms are possible, reduced focus on internal bodily states (e.g., emotions) in alexithymia may result in increases in the salience of exteroceptive stimuli, and therefore hypersensitivity. Difficulties identifying bodily states may also result in hyposensitivity due to difficulties with perception, or conversely hypersensitivity due to differences between actual and expected bodily states. Furthermore, if alexithymia results from, or causes, atypical interoception, external stimulation may be misclassified as pain, resulting in hypersensitivity, or as another less salient interoceptive signal, resulting in hyposensitivity to external stimulation (see [7] for discussion). Consistent with this hypothesis, alexithymia has been associated with sensory symptoms across several studies [37, 38], including in autistic individuals [39, 40], and has been associated with atypicalities in the processing of internal bodily states (‘interoception’; [7, 41]). Like autism, many conditions in which alexithymia is highly prevalent (e.g., ADHD; Schizophrenia; [42, 43]) commonly show sensory symptoms [24, 25]. Just as for emotional symptoms [33–35], sensory symptoms in autism may be caused by co-occurring alexithymia, not by autism per se; hence the heterogeneity seen in symptoms across autistic individuals.

As such, the aim of this twin study was to investigate any shared aetiology between autism, sensory sensitivity and alexithymia by comparing adolescent MZ versus DZ twin resemblances across traits (“cross-twin cross-trait”). For alexithymia, we focused on difficulties identifying feelings, as this facet is most likely to be linked to sensory symptoms [38]. Specifically, we sought to replicate findings of a strong genetic association between autism and sensory sensitivity, and for the first time explore the aetiological factors underlying phenotypic relationships between 1) alexithymia and autism and 2) alexithymia and sensory symptoms. Importantly, we also sought to examine the contribution of autism to the expected relationship between alexithymia and sensory symptoms and the contribution of alexithymia to the expected genetic association between autism and sensory sensitivity. With respect to the hypothesised association between alexithymia and sensory symptoms, if controlling for the variance in alexithymia associated with autism has no effect on the genetic correlation between alexithymia and sensory symptoms, this would suggest that alexithymia and sensory symptoms share genetic factors independent of autism. Conversely, if controlling for autism reduces the association between alexithymia and sensory symptoms, or results in the association becoming non-significant, this would suggest that autism, alexithymia and sensory symptoms share aetiological factors, or that associations between alexithymia and sensory symptoms are simply an artefact of the genetic correlation between alexithymia and sensory symptoms, and between alexithymia and autism. With respect to the genetic association between autism and sensory symptoms, if controlling for variance in sensory symptoms associated with alexithymia reduces the genetic correlation between autism and sensory symptoms, this suggests that the aetiological factors are shared between autism, alexithymia and sensory symptoms. If controlling for alexithymia results in the genetic correlation between autism and sensory symptoms becoming non-significant, this would suggest that there is no distinct contribution of autism to sensory symptoms beyond that shared with, or due to, alexithymia.

## Method

### Participants

This project utilised data collected in the Social Relationships Study (SR Study; [12]), a subsample of the Twins Early Development Study (TEDS; [44]), selected after autism screening. TEDS is a representative population-derived cohort of all twins born in England and Wales between January 1994 and December 1996. 412 families were eligible for inclusion in the SR Study based on at least one twin scoring ≥ 15 on the Childhood Autism Spectrum Test (CAST) at age 8 or having an autism diagnosis. Of these, 230 were retained on the basis that one or more twin met Development And Well-Being Assessment (DAWBA; [45]) telephone interview criteria for autism. Additionally, five families were identified from mail-outs to psychiatrists.

Of these 235, 106 could not be contacted, declined to participate, or only completed questionnaires. This left 129 families with at least one twin likely to meet criteria for autism who were visited at home, where gold-standard diagnostic assessments were conducted. 80 comparison families, from TEDS, in which both twins scored <12 on the CAST, were also recruited. These were age, sex, zygosity and socioeconomic status (SES) matched to the main sample. This resulted in a sample of 209 twin pairs (see Supplementary Figure S1 for details; [12]). Zygosity information was collected via parental questionnaires, and validated by DNA testing, which showed the questionnaire had 95% accuracy. For the present study, two families were excluded following home assessment: one because neither twin met diagnostic criteria for autism, and the other due to difficult family circumstances compromising diagnostic accuracy. This left 207 twin pairs with valid Best Estimate Diagnosis (BED) data, resulting in a final sample of 55 MZ pairs and 152 DZ pairs. Mean age at assessment was 13.2 years and the sample was 69% male. To ensure unbiased results, we made use of all available raw data using maximum likelihood estimation (see Results). For full details of participant numbers see Supplemental materials S2.

### Procedure

Ethical approval was granted by the Institute of Psychiatry Ethics Committee and written parental consent was obtained. All methods were performed in accordance with relevant guidelines and regulations. Trained researchers visited families at home between 2007 and 2009. Twin pairs likely to meet criteria for autism were administered the Autism Diagnostic Observation Schedule – Generic (ADOS-G; [46]), and parents were administered the Autism Diagnostic Interview – Revised (ADI-R; [47]), which together are seen as gold-standard diagnostic instruments for autism and were used to obtain a BED (see below). The entire sample (including the comparison group) also completed a battery of cognitive assessments, including the Wechsler Abbreviated Scale of Intelligence (WASI; [48]). All parents completed the Short Sensory Profile (SSP; [49]) and the *uninsightful* subscale of the Observer Alexithymia Scale (OAS; [50]).

### Measures

#### Best estimate diagnosis (BED)

Information from DAWBA, ADI-R, ADOS-G and clinical reports were reviewed by researchers blinded to zygosity. In cases with inconsistencies across these measures (N = 89), expert clinicians reviewed all available information in order to assign a BED (for details see [12]).

#### Observer alexithymia scale (OAS)

The OAS [50] is a 33-item questionnaire designed to be completed by individuals who know the participant well. In this study, only subscale 2 (the *uninsightful* subscale) was used, which comprises 8 items designed to capture lack of insight into personal feelings, a key feature of alexithymia that is most likely to be linked to sensory symptoms [38]. Whilst questions are typically rated on a 4-point Likert scale, the scale was altered to a 5-point scale ranging from 0 (strongly disagree) to 4 (strongly agree) to maintain consistency in questionnaire responding. For the OAS, internal consistency has been reported to be 0.75–0.78 across samples with test-retest reliability *r* = 0.87 over two weeks [50]. In our sample, despite amending the response scale internal consistency was excellent (α = 0.88).

#### Short sensory profile (SSP)

The Short Sensory Profile [51] is an abbreviated parent-report version of the Sensory Profile, comprising the 38 items exhibiting the most discriminative power in measurement of atypical sensory processing. Parents report using a 5-point Likert scale denoting the frequency with which their child exhibits certain behaviours. Scores are summed to produce a total score, with higher scores representing greater sensory symptoms. In our sample, internal consistency for the SSP was excellent (α = 0.94).

#### IQ

Full-scale IQ was obtained from administering all four subtests of the WASI [48]. Scores for non-verbal children, and those not able to attempt the WASI, were imputed using a composite of their verbal age scores on the British Picture Vocabulary Scale (BPVS; [52]) and scores on Raven’s Coloured Progressive Matrices [53].

### Statistical analyses

#### Data handling and preparation

From the sample of 207 individuals, complete parent report data was available for 166 twin pairs (80.2%). Total scores for each continuous scale were computed for each participant, with missing data imputed (~5% had at least one item missing for the OAS; ~8% for the SSP). As noted, we made use of all available data by using raw maximum likelihood estimation to avoid biased results (see Supplement S2 for full details of available data). Preparation for twin modelling was completed in R version 3.0 (R Core [54]). All questionnaires were adjusted for sex, age and IQ. SSP total scores were also log transformed prior to analysis due to positive skew.

#### Phenotypic correlations

Phenotypic correlations and 95% confidence intervals (CIs) were calculated for all bivariate relationships between the traits of interest, after controlling for sex, age, IQ and relatedness. Those that showed a significant phenotypic correlation were used for bivariate twin model fitting.

#### Bivariate twin model fitting

Analyses were conducted in R, using the OpenMx package [55] for structural equation modelling. A series of separate bivariate twin modelling analyses were performed in order to test the aetiological factors underlying the relationship between: 1) alexithymia (OAS) and autism (BED); 2) sensory symptoms (SSP) and alexithymia, both before and after controlling for autism; 3) sensory symptoms and autism, both before and after controlling for alexithymia. Model 2 controlled for BED by controlling for variance in alexithymia explained by autism. Whilst ideally a similar approach would have been employed for Model 3 - controlling for variance in autism explained by alexithymia - due to the selected nature of the sample with regard to the BED variable this was not possible. As such, in Model 3 we examined the relationship between sensory symptoms and autism, both before and after controlling for variance in sensory symptoms explained by alexithymia. This model allows us to determine whether there is a distinct pattern of sensory symptoms in autism that is unaccounted for by alexithymia.

Analyses involving BED used a joint continuous and ordinal method, assuming a threshold liability model underlying autism. BED was entered as an ordinal variable with three levels: unaffected; broad-spectrum; autism. Thresholds for these categories were fixed to values corresponding to lifetime prevalence values of 1% for autism and 5% for broad-spectrum, reflecting estimates from Baird et al. [56]. Thresholds were fixed to account for the nature of the sample, which was selected on the basis of screening for autism.

Shared aetiology between traits of interest was investigated using bivariate twin modelling. The rationale behind this design is the same as the univariate design, however, cross-twin resemblance is calculated across two traits (i.e. variable 1-twin 1 with variable 2-twin 2, and variable 2-twin 1 with variable 1-twin 2). These are then compared between zygosity groups. If the MZ cross-twin cross-trait correlation is higher than that of the DZ pairs, this indicates a significant genetic (A) component to the covariance. If MZ twins’ cross-trait resemblance is less than twice that of DZ twins, this indicates a significant role for common environment (C). Finally, if MZ twins resemble each other less than 100% across traits, this indicates a significant role for unique environment (E). See Supplemental Materials S3 and S4 for specification of twin correlations and bivariate ACE models, respectively.

## Results

### Phenotypic correlations

Phenotypic correlations between measures after controlling for age, sex, IQ (all previously related to the measures of interest; [10, 57, 58] and genetic relatedness, were substantial and significant; BED was correlated with OAS (r = 0.40, CI: 0.31/0.49) and SSP (r = 0.45, CI: 0.35/0.54), and OAS and SSP were also correlated (r = 0.60, CI: 0.51/0.67).

#### Model 1: alexithymia (OAS) and autism (BED)

##### Twin correlations

Within- and cross-trait twin correlations for OAS and BED are presented in Table 1, Panel A. For BED (as reported in [12]), the MZ correlation was around twice as high as DZ correlation, suggesting a significant additive genetic influence on BED, and no significant shared environmental influence. For OAS, the MZ correlation was more than twice that of the DZ correlation, suggesting a non-additive genetic contribution and again, little contribution from shared environment. The model estimated a moderate phenotypic correlation of 0.40 between the traits.

**Table 1:** A. MZ and DZ within- and cross-trait correlations for OAS and BED. B. Standardised estimates of the bivariate ACE model for OAS and BED.

Panel A			
	r_ph_	r_MZ_	r_DZ_
OAS		**0.88 (0.79/0.92)**	**0.35 (0.20/0.49)**
BED		**0.91 (0.85/0.95)**	**0.47 (0.37/0.56)**
Cross-trait	**0.40 (0.31/0.49)**	**0.37 (0.26/0.46)**	0.09 (−0.01/0.20)

Panel B			
		OAS	BED
Standardised Estimates	h^2^	**0.84 (0.67/0.91)**	**0.82 (0.72/0.85)**
	c^2^	0.04 (0.00/0.16)*	**0.08 (0.03/0.24)**
	e^2^	**0.13 (0.09/0.22)**	**0.09 (0.05/0.16)**
	r_A_	**0.51 (0.37/0.70)**	
	r_C_	−1.00 (−1.00/1.00)	
	r_E_	**0.41 (0.02/0.72)**	
	r_ph_	**0.41 (0.32/0.49)**	
Part of Phenotypic Correlation	r_ph-A_	**0.42 (0.32/0.54)**	
	r_ph-C_	−0.06 (−0.13/0.04)	
	r_ph-E_	**0.04 (0.03/0.10)**	

r_ph_ denotes the phenotypic correlation estimated by the model; r_MZ_, monozygotic cross-twin correlation; r_DZ_, dizygotic cross-twin correlation; h^2^ denotes heritability component estimated by the model; c^2^, common environmental component; e^2^, unique environmental component; r_A_, additive genetic correlation; r_C_, common environmental correlation; r_E_, unique environmental correlation; r_ph_, phenotypic correlation; r_ph-A_, part of phenotypic correlation due to additive genetic influences; r_ph-C_, part of phenotypic correlation due to common environmental influences; r_ph-E_, part of phenotypic correlation due to unique environmental influences. Note that for aesthetics 2 decimal places are used for confidence intervals when assessing significance (indicated by bold text).

*denotes where a significant effect is present when a more conservative threshold is used (i.e. any value > 0).

Standardised estimates derived from the bivariate ACE model are shown in Table 1, Panel B (see Supplementary Figure S5a for path diagram). BED showed a heritability estimate of 82%, slightly lower than other estimates [12, 59]. OAS scores were highly heritable (84%), with moderate influences (13%) from non-shared environment. Since shared environmental influences were not negligible, the full ACE model was retained. The correlation between BED and OAS was mostly explained by genetic factors, with the remainder explained by unique environmental contributions. We also examined the association between BED and OAS after controlling for SSP (see Supplementary Figure S5b for path diagram). Though caution is required as it is possible that the model may not have reached full optimisation (which sometimes occurs with ordinal data - see Discussion for elaboration), after controlling for SSP, BED and OAS were significantly associated (0.17 CI: 0.07/0.27), with this association only attributable to genetic factors.

#### Model 2: alexithymia (OAS) and sensory symptoms (SSP)

##### Twin correlations

The correlational model for OAS and SSP (Table 2A) indicated that the cross-trait phenotypic correlation was 0.60 before controlling for BED, with a 2:1 rMZ:rDZ ratio indicating this relationship to be genetic in nature.

**Table 2:** A: MZ and DZ within- and cross-trait correlations for SSP and OAS. B. Standardised estimates of the bivariate ACE model for OAS and SSP, with and without controlling for BED.

Panel A						
				OAS corrected for BED		
	r_ph_	r_MZ_	r_DZ_	r_ph_	r_MZ_	r_DZ_
SSP		**0.86 (0.78/0.91)**	**0.53 (0.38/0.65)**		**0.86 (0.78/0.91)**	**0.53 (0.37/0.65)**
OAS		**0.89 (0.82/0.93)**	**0.33 (0.15/0.48)**		**0.86 (0.77/0.91)**	**0.39 (0.22/0.53)**
Cross-trait	**0.60 (0.51/0.67)**	**0.51 (0.40/0.60)**	**0.23 (0.08/0.36)**	**0.35 (0.23/0.46)**	0.**30 (0.18/0.42)**	**0.15 (0.01/0.29)**

Panel B					
		Before correcting OAS for BED		After correcting OAS for BED	
		OAS	SSP	OAS	SSP
Standardised Estimates	**h**^**2**^	**0.88 (0.18/0.93)**	**0.61 (0.36/0.86)**	**0.86 (0.63/0.91)**	**0.67 (0.40/0.90)**
	c^2^	0.01 (0.00/0.14)*	**0.25 (0.01/0.47)**	0.00 (0.00/0.21)*	0.20 (0.00/0.44)*
	**e**^**2**^	**0.11 (0.07/0.19)**	**0.14 (0.09/0.23)**	**0.14 (0.09/0.24)**	**0.14 (0.09/0.22)**
	**r**_**A**_		**0.62 (0.46/0.74)**		**0.37 (0.13/0.57)**
	**r**_**C**_		1.00 (−1.0/1.0)		1.0 (−0.88/1.0)
	**r**_**E**_		**0.72 (0.53/0.84)**		**0.34 (0.03/0.59)**
	**R**_**ph**_		**0.60 (0.35/0.67)**		**0.35 (0.23/0.46)**
Part of Phenotypic Correlation	**R**_**ph-A**_		**0.45 (0.27/0.59)**		**0.28 (0.08/0.44)**
	**R**_**ph-C**_		0.06 (−0.06/0.22)		0.03 (−0.11/0.21)
	**R**_**ph-E**_		**0.09 (0.05/0.16)**		0.05 (0.00/0.11)*

r_ph_ denotes the phenotypic correlation estimated by the model; r_MZ_, monozygotic cross-twin correlation; r_DZ_, dizygotic cross-twin correlation. h^2^ denotes heritability component estimated by the model; c^2^, common environmental component; e^2^, unique environmental component; r_A_, additive genetic correlation; r_C_, common environmental correlation; r_E_, unique environmental correlation; r_ph_, phenotypic correlation; r_ph-A_, part of phenotypic correlation due to additive genetic influences; r_ph-C_, part of phenotypic correlation due to common environmental influences; r_ph-E_, part of phenotypic correlation due to unique environmental influences. Note that for aesthetics 2 decimal places are used for confidence intervals when assessing significance (indicated by bold text).

*denotes where a significant effect is present when a more conservative threshold is used (i.e. any value > 0).

**Fig. 1:**
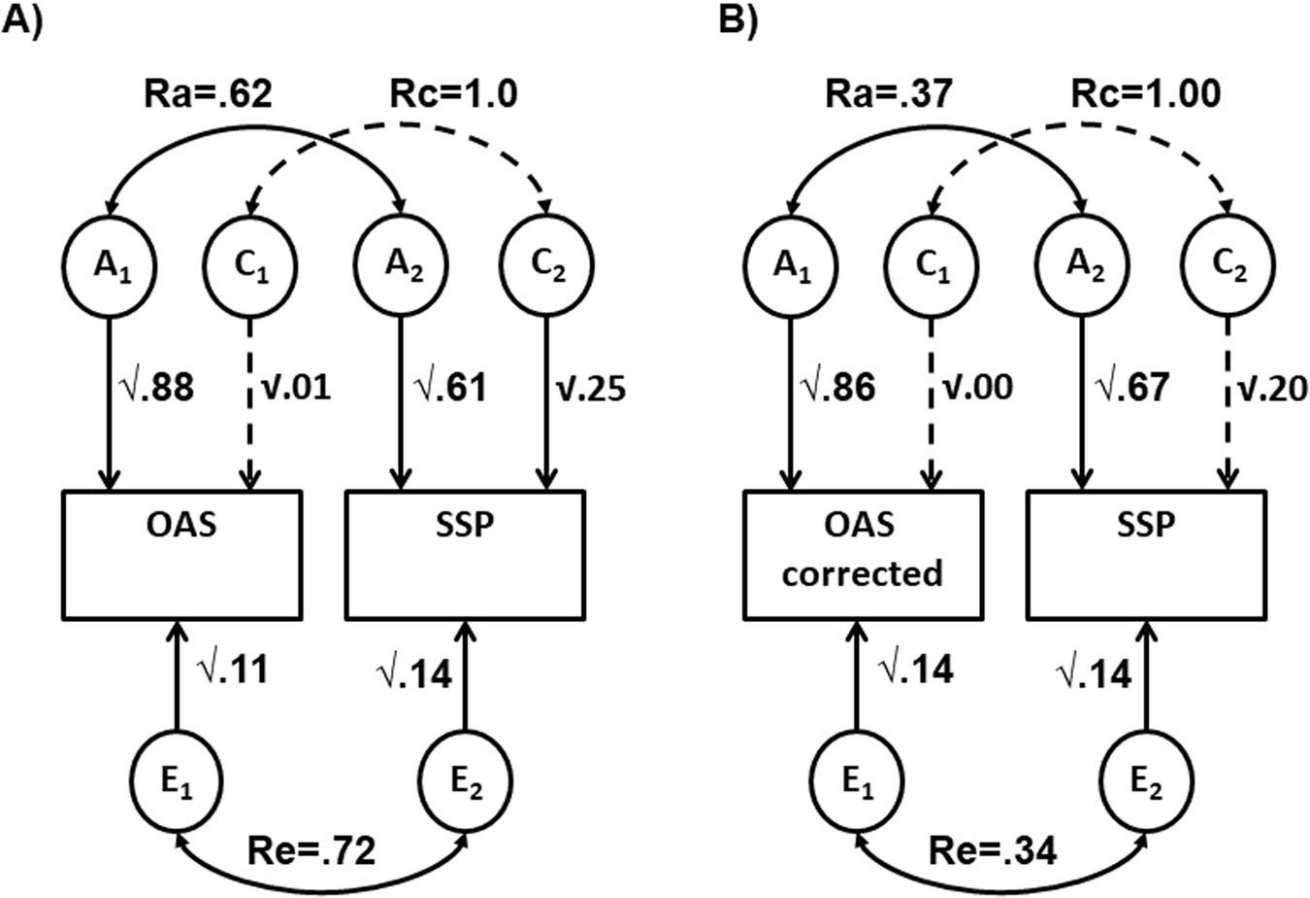
Path diagram showing results of bivariate ACE modelling for OAS and SSP. **A** Before controlling for BED; **B** after controlling for BED. SSP denotes Observer Alexithymia Scale, OAS Observer Alexithymia Scale, rA additive genetic correlation, rC common environmental correlation, rE unique environmental correlation. Dashed lines indicate non-significant estimates; solid lines, significant estimates.

The correlation between SSP and OAS (uncorrected for BED) of 0.60 was 75% due to genetic factors (R_ph-A_ = 0.45). After controlling for BED, the phenotypic correlation dropped to 0.35 but remained significant. Importantly, the genetic correlation between OAS and SSP also remained significant, with the additive genetic component remaining the largest contributor to shared variance (0.28; 80%; see Table 2 Panel B). The contribution from shared environmental factors for OAS and SSP did not reach significance in either analysis, however, this sample may have lacked power to detect this contribution. The remaining covariance was explained by non-shared environmental factors. Figure 1 displays these results as path diagrams.

#### Model 3: sensory symptoms (SSP) and autism (BED)

##### Twin correlations

For SSP, rDZ was more than half that of rMZ (Table 3A), suggesting that common environmental, as well as additive genetic factors, contribute to its variance. The rMZ:rDZ ratio for the cross-trait correlations between BED and SSP is 2:1, suggesting additive genetic factors largely explain the bulk of their covariance. However, when OAS scores were regressed out of SSP, much of its phenotypic correlation with BED was removed, despite the correlation remaining significant. Further, the MZ cross-trait cross-twin correlation dropped to 0.06, rendering it similar to the DZ cross-trait cross-twin correlation of 0.10.

**Table 3:** A: MZ and DZ within- and cross-trait correlations for SSP and BED. B. Standardised estimates of the bivariate ACE model for BED and SSP, with and without controlling for OAS.

Panel A						
				SSP corrected for OAS		
	r_ph_	r_MZ_	r_DZ_	r_ph_	r_MZ_	r_DZ_
SSP		**0.84 (0.75/0.90)**	**0.56 (0.42/0.66)**		**0.87 (0.78/0.92)**	**0.49 (0.34/0.61)**
BED		**0.92 (0.85/0.95)**	**0.47 (0.38/0.56)**		**0.92 (0.86/0.96)**	**0.47 (0.37/0.56)**
Cross-trait	**0.45 (0.35/0.54)**	**0.37 (0.26/0.47)**	**0.20 (0.10/0.31)**	**0.13 (0.03/0.23)**	0.06 (−0.05/0.17)	0.10 (−0.01/0.21)

Panel B					
		Before correcting SSP for OAS		After correcting SSP for OAS	
		BED	SSP	BED	SSP
Standardised Estimates	**h**^**2**^	**0.87 (0.67/0.92)**	**0.57 (0.32/0.85)**	**0.85 (0.80/0.94)**	**0.73 (0.49/0.90)**
	**c**^**2**^	**0.03 (0.02/0.16)**	**0.27 (0.01/0.48)**	**0.06 (0.01/0.22)**	0.14 (0.00/0.32)*
	**e**^**2**^	**0.08 (0.07/0.13)**	**0.16 (0.10/0.25)**	**0.08 (0.04/0.13)**	**0.13 (0.13/0.21)**
	**r**_**A**_		**0.47 (0.23/0.69)**		−0.05 (−0.28/0.11)
	**r**_**C**_		0.40 (−1.0/1.0)		1.0 (−1.0/1.0)
	**r**_**E**_		**0.67 (0.30/0.90)**		**0.64 (0.25/0.88)**
	**R**_**ph**_		**0.45 (0.35/0.54)**		**0.12 (0.02/0.22)**
Part of Phenotypic Correlation	**R**_**ph-A**_		**0.33 (0.16/0.50)**		−0.04 (−0.10/0.10)
	**R**_**ph-C**_		0.04 (−0.11/0.14)		0.09 (−0.01/0.21)
	**R**_**ph-E**_		**0.08 (0.05/0.12)**		**0.07 (0.03/0.12)**

r_ph_ denotes the phenotypic correlation estimated by the model; r_MZ_, monozygotic cross-twin correlation; r_DZ_, dizygotic cross-twin correlation. h^2^ denotes heritability component estimated by the model; c^2^, common environmental component; e^2^, unique environmental component; r_A_, additive genetic correlation; r_C_, common environmental correlation; r_E_, unique environmental correlation; r_ph_, phenotypic correlation; r_ph-A_, part of phenotypic correlation due to additive genetic influences; r_ph-C_, part of phenotypic correlation due to common environmental influences; r_ph-E_, part of phenotypic correlation due to unique environmental influences. Note that for aesthetics 2 decimal places are used for confidence intervals when assessing significance (indicated by bold text).

*denotes where a significant effect is present when a more conservative threshold is used (i.e. any value > 0).

**Fig. 2:**
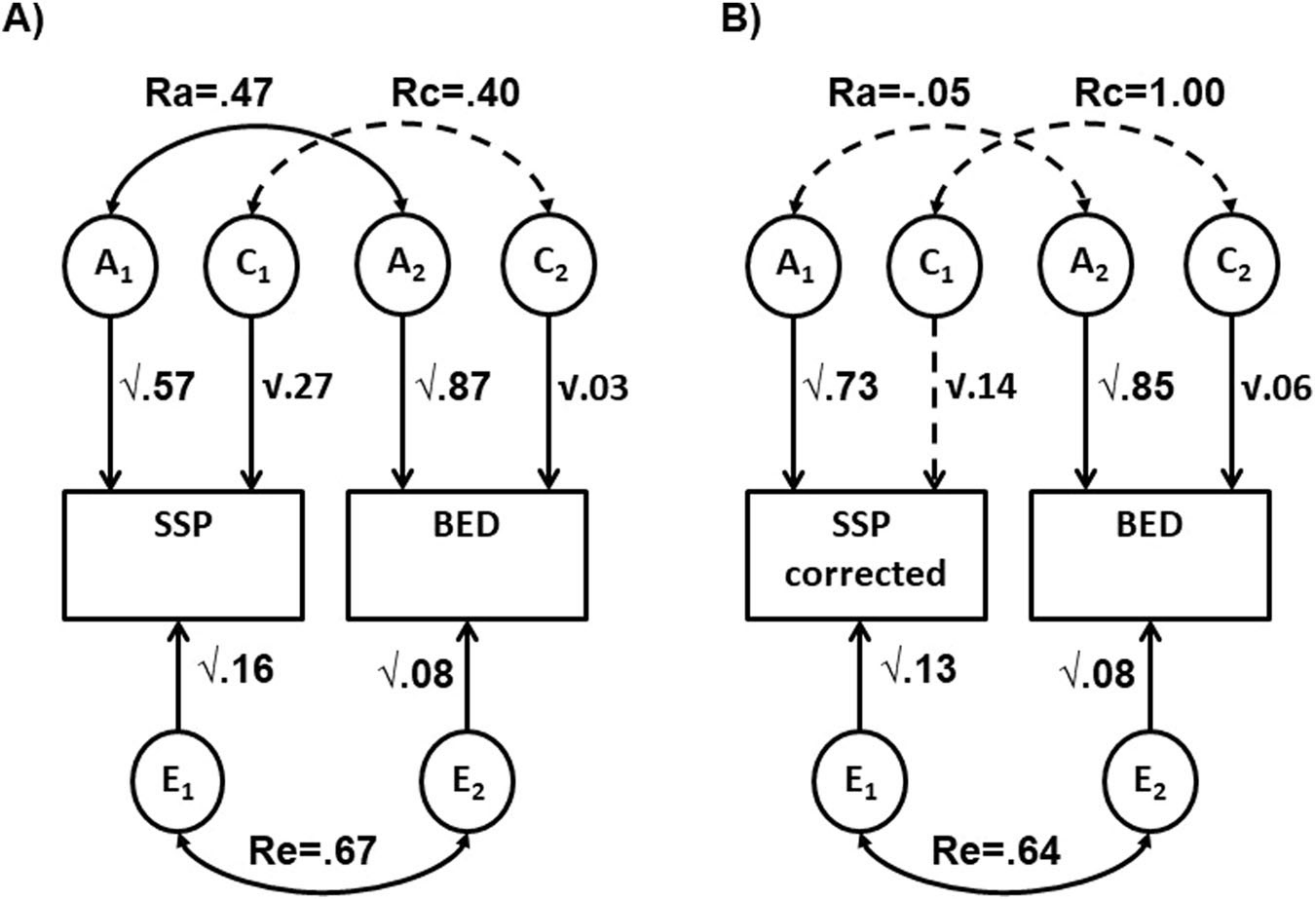
Path diagram displaying results of bivariate ACE modelling for SSP and BED. **A** Before controlling for OAS; **B** after controlling for OAS. SSP denotes Short Sensory Profile, BED Best Estimate Diagnosis, rA additive genetic correlation, rC common environmental correlation, rE unique environmental correlation. Dashed lines indicate non-significant estimates; solid lines, significant estimates.

Standardised estimates from the bivariate ACE model between SSP and BED (Table 3B) show similar outcomes for BED as in the OAS-BED model. SSP scores are moderately heritable (57%) with significant influences from both shared environmental (27%) and unique environmental (16%) sources. 73% of the phenotypic correlation between BED and SSP was explained by overlapping genetic factors, with some contribution from unique environment (17%). When OAS was controlled for, estimates for h^2^ increased slightly for SSP with c^2^ becoming non-significant. However, importantly the genetic correlation (r_A_) between SSP and BED was reduced and the genetic component to their covariance became non-significant. Figure 2 illustrates the ACE model results in path diagram form.

## Discussion

This study investigated the shared aetiology of autism, alexithymia and sensory symptoms to elucidate the genetic association between autism and sensory symptoms after accounting for alexithymia, and between alexithymia and sensory symptoms after accounting for autism. As expected, associations were observed between all factors with all bivariate associations largely explained by genetic factors. Importantly, controlling for the contribution of alexithymia to sensory symptoms reduced the association between autism and sensory symptoms and the genetic correlation was no longer significant. In contrast, whilst controlling for the contribution of autism to alexithymia reduced the phenotypic correlation between alexithymia and sensory symptoms, this association remained significant, and the proportion of variance explained by genetic factors remained constant. The implications of these results are discussed below.

Heritability estimates for each trait were mostly consistent with prior research. Autism was highly heritable (~82%; [12]), as were sensory symptoms (~61%; [16–18]), though heritability of alexithymia (~88%) was higher than prior estimates [27–29]. For alexithymia, differences across studies may reflect methodological differences such as the use of parent-report, self-report questionnaires or interviews, as well differences in the symptoms captured by measures.

Turning to the associations between traits, as expected autism and alexithymia were moderately associated, consistent with the overlap reported in the literature [30]. This phenotypic association was exclusively genetic in origin, with very low and non-significant estimates for environmental contributions, which is consistent with a common genetic contribution to these traits. However, it is important to note that not all heritability is shared. This is consistent with research suggesting that whilst alexithymia and autism commonly co-occur they are separate conditions [32], with our results indicating the existence of genes that influence each trait independently of the other. As this genetic association remained after controlling for sensory symptoms, this suggests that alexithymia and autism share genetic factors beyond those contributing to sensory symptoms, consistent with evidence that alexithymia may contribute to other features of autism (e.g., emotional symptoms; [33, 35, 36]). However, some caution is required in interpreting these results, as full optimisation may not have been achieved due to the use of ordinal data.

As expected, autism and sensory processing were also moderately associated, consistent with prior work examining phenotypic associations between these traits [5]. As has previously been observed, this association was mostly explainable by genetic factors [18, 19]. Importantly, however, controlling for the contribution of alexithymia to sensory symptoms reduced the association between autism and sensory processing. These results suggest that no genetic association remains between sensory processing and autism after controlling for alexithymia, but it should be noted that we examined the effect of controlling for the contribution of alexithymia to sensory symptoms (and not the contribution of alexithymia to autism). These results indicate both that there are shared aetiological factors between autism, alexithymia and sensory symptoms, and that there is no distinct genetic contribution of autism to sensory symptoms.

Finally, a high correlation between sensory symptoms and alexithymia was observed, consistent with phenotypic reports [37, 38, 40], with results showing for the first time that there is a substantial degree of shared genetic variance. Crucially, this phenotypic and genetic relationship remained significant after controlling for autism, with a reduction only observed for the phenotypic association; the amount of variance attributable to genetics remained constant. This suggests that alexithymia and sensory processing share genetic factors, independent of those that increase the likelihood of autism. As such, although alexithymia and sensory symptoms commonly co-occur with autism (potentially due to a degree of shared genetic liability), they are also independent from autism.

Taken together, these findings suggest that alexithymia may explain individual differences in sensory processing in the autistic population as it does variation in emotional processing [33, 35, 36]. Given reports that alexithymia may influence autism diagnosis [60], and that sensory symptoms are now included in the autism diagnostic criteria [8], these results suggest a need to consider the influence of alexithymia both when diagnosing autism and providing support. Autism with co-occurring alexithymia and/or sensory processing difficulties may represent a specific subtype of autism with homogenous genetic antecedents, which may respond in a similar way to interventions. Examining the specific facets to which alexithymia contributes (given that both sensory symptoms and autism are multifaceted; [61, 62]) may prove useful for understanding this link further. As evidence suggests alexithymia is observed across multiple conditions [63], extending the present approach to examine the contribution of alexithymia to sensory symptoms in other conditions (e.g., ADHD) would shed light on whether alexithymia can be considered a transdiagnostic factor associated with atypical sensory symptoms across multiple conditions.

Despite the utility of these data in understanding the relationship between autism, sensory symptoms and alexithymia, several limitations must be acknowledged. First, this study may have lacked power to detect small, yet potentially important, influences of the shared environment, which behavioural evidence suggests may play a role in the manifestation of sensory symptoms in autism [64]. Power also prevented investigation of sex differences, which are the subject of much recent autism research [65]. Relatedly, despite efforts to collect full-sample data, not all families completed questionnaires. As a greater proportion of missing data was from autistic participants, this may have reduced scope for detecting associations between alexithymia, sensory symptoms and autism. Second, only parent-report data was utilised, and as such it is possible that reporter bias may have affected the data, as one parent is likely to have completed questionnaires for both twins. This means that cross-twin and cross-trait correlations could have been inflated due to similar interpretations and response style to questions. However, whilst this may have inflated the phenotypic correlations for sensory symptoms and alexithymia, it is unlikely to have changed the ratio between MZ and DZ twins. It should also be noted that parent-report and self-report measures of alexithymia do not always agree [66, 67], and therefore different results may be observed using self-report measures. It is difficult to know which measure should be considered a more accurate measure of alexithymia as parent-reports may underestimate alexithymic characteristics due to their reliance on observable behaviour, but individuals with high levels of alexithymia may lack insight into their emotional difficulties. Future research should therefore aim to replicate these results using data from multiple informants. Third, only the uninsightful subscale of the OAS was examined in this study. Whilst this subscale is most likely to be linked to sensory symptoms as it taps difficulties identifying feelings, these data cannot speak to the relationship between sensory symptoms and other facets of alexithymia (i.e., difficulties describing feelings or externally orientated thinking). Fourth, the OAS response scale was slightly altered for consistency with the other scales. However, this is unlikely to have greatly affected the measurement of alexithymia, as internal consistency was excellent. Fifth, for the model examining the relationship between OAS and BED after controlling for SSP (see Supplement) full optimisation may not have been achieved. Although the solution is often correct, it is difficult to know whether and how the results may be affected as optimisation of all parameters occurs simultaneously; for example, if heritability decreases, then either shared or non-shared environment would increase, and vice versa. As such, whilst caution is required when interpreting these specific results, as this analysis did not relate to the primary questions this does not challenge the main conclusions. Finally, due to the ordinal nature of the BED variable it was not possible to examine the impact of controlling for the contribution of alexithymia to autism when examining the relationship between autism and sensory symptoms. As such, it is not possible from these analyses wholly to determine whether alexithymia fully explains the genetic association between autism and sensory symptoms. Future research that assesses the relationship between autistic traits, alexithymia and sensory symptoms may prove useful for elucidating these relationships further.

Despite limitations, these data provide novel insights into the aetiological relationships between alexithymia, sensory symptoms and autism, suggesting that 1) alexithymia and sensory symptoms share aetiology that is not accounted for by their association with autism, and 2) alexithymia may explain the genetic relationship between sensory symptoms and autism, indicating there may be no distinct contribution of autism to sensory symptoms. Further research examining the contribution of alexithymia to sensory processing difficulties across conditions will shed light on whether alexithymia can be considered a transdiagnostic factor marking atypical sensory processing.

## Supplementary information

Accepted supplementary material without tracked changes or highlights

## Data Availability

Data is available on request.

## Footnotes

1 We use the term ‘autistic’ and identity-first language as this is endorsed by the majority of the autistic community (see [1]).

## Code availability

All code is available upon reasonable request.
