# Supplementary material for: Alexithymia may explain the genetic relationship between autism and sensory sensitivity
Source: Transl Psychiatry. 2025 Mar 5;15:75. doi: 10.1038/s41398-025-03254-1 (PMC11882979; doi:10.1038/s41398-025-03254-1)
Supplement: Supplementary file 1 — Accepted supplementary material without tracked changes or highlights [file 41398_2025_3254_MOESM1_ESM.docx]

**Supplementary Information and Results**

**[S1] Selection stages**


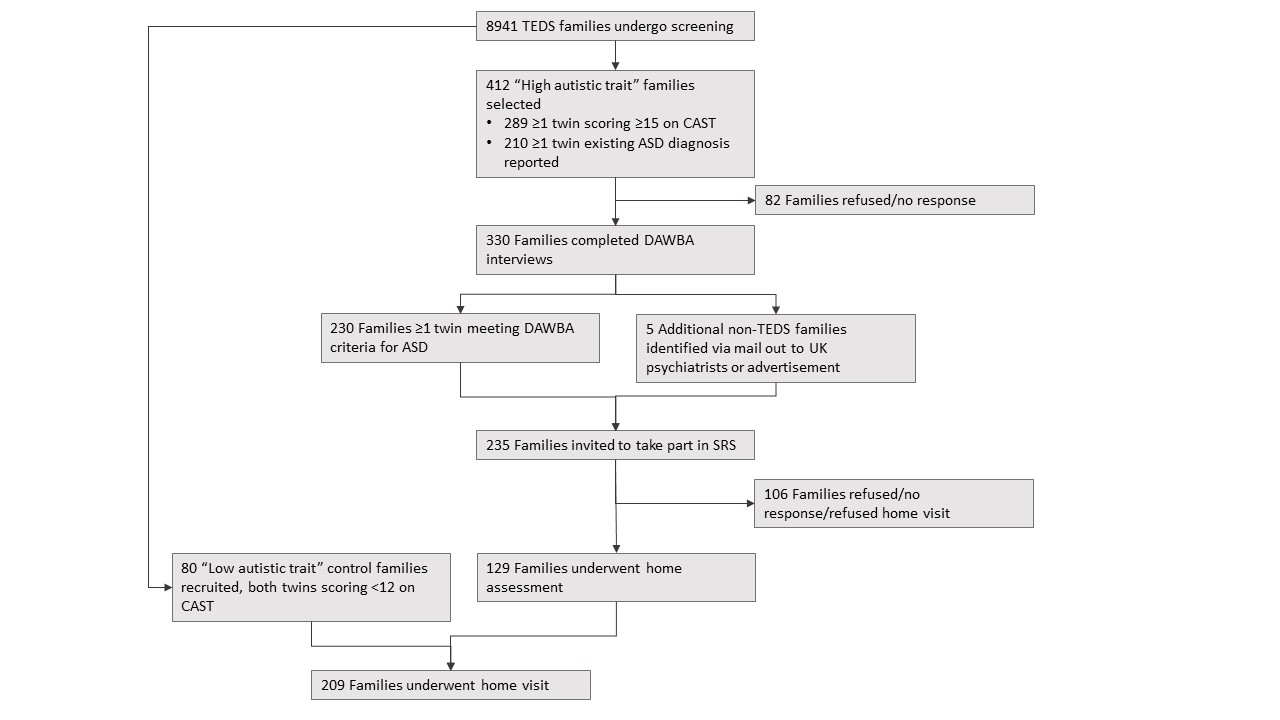


| **Figure S1. Selection stages for inclusion in the Social Relationships Study. Adapted from Colvert *et al*. (Colvert et al., 2015)(Colvert et al., 2015) (Figure 1).**  TEDS denotes the Twins Early Development Study; CAST, Childhood Autism Spectrum Test; ASD, Autism Spectrum Disorder; DAWBA, Development and Well-being Assessment; SRS, Social Relationships Study. |
| --- |

##### **[S2] Participant numbers for raw data**

|  |  | MZ | | | DZ | | |
| --- | --- | --- | --- | --- | --- | --- | --- |
|  | Total | BED_01 | BED_02 | BED_03 | BED_01 | BED_02 | BED_03 |
| BED | 414 | 61 | 9 | 42 | 172 | 31 | 99 |
| FSIQ | 385 | 59 | 8 | 33 | 168 | 29 | 82 |
| OAS | 340 | 51 | 6 | 33 | 146 | 23 | 73 |
| SSP | 314 | 48 | 5 | 26 | 144 | 24 | 60 |

*Table S2. Depicting available data across all variables employed. MZ = Monozygotic; DZ = Dizygotic; BED= Best Estimate Diagnosis; BED_01 = unaffected and controls; BED_02 = Broad type; BED_03 = ASD; FSIQ = Full scale IQ. OAS = Observer alexithymia scale (uninsightful subscale); SSP = Short Sensory profile*

##### **[S3] Twin Correlations: specification and interpretation**

Twin Correlations: specification and interpretation

Correlations between first-born twins and second-born twins (categorised according to zygosity), controlling for relatedness of observations, were estimated within and across traits. This correlational model used constraints to obtain a reduced number of statistics, thereby facilitating interpretation (see Supplementary Table S3 for an example). To this end, appropriate elements of the standardized correlation matrix were labelled identically in order to constrain them to the same value. The within-twin cross-trait correlations (labelled r_ph_) are assumed to be the same, regardless of birth order and zygosity group. According to the model, cross-twin within-trait correlations differ depending on trait and zygosity and thus are assigned different labels: r_MZ_11; r_MZ_22; r_DZ_11; r_DZ_22. The cross-twin cross-trait correlations (OAS1-SSP2 and OAS2-SSP1 in the example) are modelled to be the same regardless of birth order, but different for each zygosity group, and hence labelled r_MZ_21 and r_DZ_21 for MZ and DZ groups, respectively.

Standard deviations for each trait are assumed to be equal across birth order and zygosity, thus are labelled SD1 and SD2 (for BED, SD is constrained to equal 1 in accordance with the liability threshold model; See Table S3). Likewise, means for each trait are constrained to be the same across birth order and zygosity. By comparing the resulting MZ and DZ correlations, the relative contributions from genetic (A), shared environmental (C) and non-shared environmental (E) sources are indicated according to the logic outlined above. A, C and E components to variance in traits, and covariance between traits, were then formally estimated in a series of bivariate genetic models. These models also yielded confidence intervals for each parameter.

| **Table S3. Example matrices showing constraints on parameters used in bivariate twin modelling.** | | | | | | | | | | |
| --- | --- | --- | --- | --- | --- | --- | --- | --- | --- | --- |
| MZ Correlation Matrix | | | | |  | DZ Correlation Matrix | | | | |
|  | OAS1 | SSP1 | OAS2 | SSP2 |  |  | OAS1 | SSP1 | OAS2 | SSP2 |
| OAS1 | 1 |  |  |  |  | OAS1 | 1 |  |  |  |
| SSP1 | r_ph_ | 1 |  |  |  | SSP1 | r_ph_ | 1 |  |  |
| OAS2 | r_MZ_11 | r_MZ_21 | 1 |  |  | OAS2 | r_DZ_11 | r_DZ_21 | 1 |  |
| SSP2 | r_MZ_21 | r_MZ_22 | r_ph_ | 1 |  | SSP2 | r_DZ_21 | r_DZ_22 | r_ph_ | 1 |
| MZ Standard Deviation Matrix | | | | |  | DZ Standard Deviation Matrix | | | | |
|  | OAS1 | SSP1 | OAS2 | SSP2 |  |  | OAS1 | SSP1 | OAS2 | SSP2 |
| OAS1 | SD1 |  |  |  |  | OAS1 | SD1 |  |  |  |
| SSP1 |  | SD2 |  |  |  | SSP1 |  | SD2 |  |  |
| OAS2 |  |  | SD1 |  |  | OAS2 |  |  | SD1 |  |
| SSP2 |  |  |  | SD2 |  | SSP2 |  |  |  | SD2 |
| MZ denotes monozygotic; DZ, dizygotic; OAS1, Observer Alexithymia Scale for twin 1; OAS2, Observer Alexithymia Scale for twin 2; SSP1, Short Sensory Profile for twin 1; SSP2, Short Sensory Profile for twin 2; r_ph_, phenotypic correlation; SD, Standard Deviation. | | | | | | | | | | |

##### **[S4] Bivariate ACE Model: specification and interpretation**

Guided by biometrical genetic theory, covariance between measures was partitioned into additive genetic (A), common environmental (C) and unique environmental (E) factors, using Cholesky decomposition (Loehlin, 1996). However, because no trait was primary to another, estimates were standardised in order to interpret correlated factors solutions. Figure S4 shows an example path diagram for this solution, whereby the genetic correlation (r_A_) is the correlation between A_1_ and A_2_ factors and the paths from these to the observed variables are given by the square root of their heritabilities. Relationships between the C factors and the E factors are derived in the same way. Contributions to phenotypic correlations (r_ph_) between the variables, from genetic (r_ph-A_), shared environmental (r_ph-C_) and unique environmental (r_ph-E_) sources were calculated via path tracing rules (see Supplementary Figure S3 for example) with the following equations: additive genetic component, r_ph-A_ = √h_1_^2^ x r_A_ x √h_2_^2^; common environmental component, r_ph-C_=√c_1_^2^ x r_C_ x √c_2_^2^; unique environmental component, r_ph-E_ =√e_1_^2^ x r_A_ x √e_2_^2^. r_ph_ is the sum of all paths from one trait to another (r_ph-A_ + r_ph-C_ + r_ph-E_). Provided these components have the same sign, they can be expressed as percentages of the total phenotypic correlation.


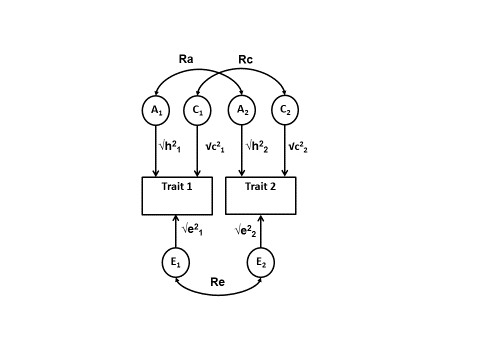


**Figure S4. Example path diagram for standardized solution of a bivariate ACE twin model**. A_1_ denotes additive genetic component for trait 1; A_2_, additive genetic component for trait 2; C_1_, common environmental component for trait 1; C_2_, common environmental component for trait 2; E_1_, unique environmental component for trait 1; E_2_, unique environmental component for trait 2; r_A_, r_C_, r_E_, cross-trait correlation between A, C and E components; √h^2^, √e^2^, √c^2^, standardised path estimates for A, C and E components of each trait. According to path tracing rules, the phenotypic correlation between the traits, r_ph_, is calculated as the sum of all legitimate paths linking Trait 1 to Trait 2.

**[S5] Path diagram showing results of bivariate ACE modelling for OAS and BED.**


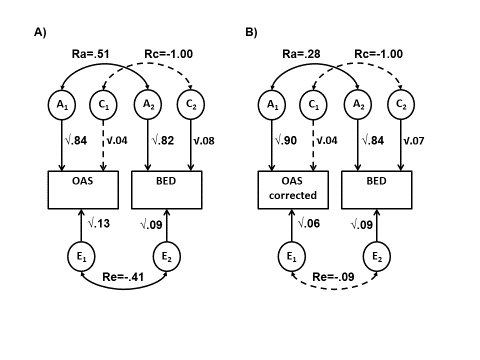


| **Figure S5. Path diagram showing results of bivariate ACE modelling for OAS and BED:**  **A) before controlling for SSP;**  **B) after controlling for SSP.**  OAS denotes Observer Alexithymia Scale; BED, Best Estimate Diagnosis; r_A_, additive genetic correlation; r_C_, common environmental correlation; r_E_, unique environmental correlation. Dashed lines indicate non-significant estimates; solid lines, significant estimates. |
| --- |
